# Brain Workers

**Published:** 1877-12

**Authors:** 


### *BRAIN WORKERS.*

Bankers, railroad managers, insurance officers, great merchants, ministers and lawyers, all suffer sooner or later from two causes—overwork of brain and underwork of body. We know well that if brain and body are both exercised, health is maintained. We know, too, that if the body is laboriously employed while the brain remains inactive, the animal tendencies increase, and intelligence wanes. We are also aware that the brain powers cannot be kept in prolonged tension by earnest and prolonged mental effort, without more or less injury, unless the body is at the same time in such a condition of active exertion or exercise as to determine the blood from the brain to other vital organs, and to the extremities. Repose is essential to mental effort; and as concentrated thought and active bodily exercise are incompatible, the serious problem for the brain-worker is this: How shall I live and preserve health of body and mind, through years of close application to my business—my banking-house, my office, my counting-room, my study—regardless of exercise?

The answer is this: there is no absolute substitute for exercise, just as there is no substitute for air or sunlight. Nothing so relieves the overwrought brain as prolonged muscular movements in the open air. Exercise of some kind is possible to all, either as a brisk walk, a horseback ride, or muscular manipulation by the strong hands of an attendant. If exercise be neglected in part, we must give more thoughtful heed to other conditions. We must not supplement vicious habits with imperfect food. The law of waste and repair is only partially understood, but there is a good deal of evidence to the fact that if our food is poor in the elements which are evolved or wasted in a certain line of labor, that description of labor soon becomes onerous, burdensome, difficult, impossible. The blacksmith wastes nitrogen; if we feed him on bread and butter—carbon—he wields his hammer but listlessly. Supply him with beef and beans, and he returns to his labor each morning, strong as a giant refreshed with sleep. The brain-worker must have his appropriate aliment also, or starvation, and the feebleness of action which always accompanies it, are certain to result, and the end of brain-starving is paralysis and imbecility, preceded often by long continued pain and misery.

In brain-starvation there is little to be derived from medicine. Business ties should be loosened or severed, and life in the open air should begin. The cure comes through change, and of all changes a change of diet from the usual to the scientific plan is the most important. The elements lacking must be supplied in food. Not in the quack mixtures advertised as "brain food," because these are merely solutions of drugs, put in bottles which bear on their labels the grossest misstatements. Our friends must not be misled into the belief that these bottled fluids are foods in any sense, even though the directions suggest that they be swallowed in milk, or tea, or coffee, or chocolate. The sufferer who needs phosphorus or iron, or other potent drug, will do well to take it under the advice of a skilled physician, instead of dosing himself with an unscientific mixture bearing a food label. The truth is, that "brain food" is not composed of stimulating drugs or nerve-excitants which temporarily elevate and permanently depress. Brain-food is blood-food, and nothing more. If our food contains, in easily assimilative form, the elements which go to make perfect blood, and if the air we breathe be pure and abundant, and if the action of the heart and lungs be encouraged by activity of the limbs—then will our blood be rich and pure, and every function of brain and nerves and muscles will be vigorously performed. The out-door laborer can make blood which will serve his purpose for a time, out of the food with which the average table is supplied; but the sedentary brain-worker who seeks greater achievements, and employs higher powers, must be provided with more refined and more highly vitalized nutriment, or sooner or later brain power wanes. When the brain is exhausted beyond the power of sleep to restore, when even sleep fails to come at our bidding, when our daily food palls upon the appetite, or fails to impart strength and vigor, without which life is burdensome, when all the instrumentalities at our command are found to be inadequate to impart energy of brain and body; and when the fear comes upon us that all is not quite as it should be—then, if we are wise, we will seek out the food-chemist, instead of the doctor and the druggist, and ask him whether the fuel which we are daily supplying to the furnace is the best possible fuel to generate the kind of force which our avocation demands. We can live after a fashion on a weak and imperfect diet, but if the food-fuel is bad for a considerable period, the machine will work but languidly after many days, and the labor which was a delight will be a burden.

Let us apply to ourselves a little of that wisdom which we employ upon our horses and cows. When they are "out of condition," we change their food and improve it. When we are out of condition in body or brain, let us make for ourselves a similar change, and let that change be directed by knowledge and scientific skill.

---

Many will ask, as many do each month, to whom shall we apply for advice and instruction in matters of diet? Our answer is, as it has been for years—to the Health Food Company of New York. They are not only the *best* authority in America on all topics connected with food-chemistry, but are, in fact, the only scientific advisers whose services are at the command of all sufferers without fee. They receive patients and the friends of patients who are too ill to call in person, and freely advise such a course as seems to be indicated. Letters addressed to them are certain to meet with a prompt and thoughtful response. Multitudes avail themselves of these precious privileges, and the uniform expression appears to be that of gratitude and satisfaction for genuine benefits experienced. The superintending and advising head of the Company is a physician of wide experience, an earnest toiler in the domain of food-chemistry. By slow and sure steps he has won the confidence and regard of our leading medical men—the heads of our colleges, hospitals, and public institutions. It is safe to say that thousands owe health and life to the researches, the wisdom, and the skill of this earnest and able physiologist, and the beneficent society over which he presides.

---

### *HOW IT WINS ITS WAY.*

Mr. Joel McComber, the great inventor and shoe manufacturer, writes us the following note, which we print with pleasure :

"Dr. E. H. Gibbs : I desire to tell your readers, in a few words, how rapidly the excellent Cold Blast Whole Wheat Flour has won its way in my family. At first I was the only consumer of the delicious bread made from this flour. All the others surrounding my table neglected this and chose the pure white loaves and biscuits. After a little my bread seemed to disappear more rapidly. A week or two later I observed that one after another were eating my bread and neglecting the old sort. Another month went by and nobody would touch the old kind, and consequently it no longer appeared on the table. We all feel better and stronger than ever before, and my own very exhausting duties do not wear me out as formerly. Our family unanimously declare this bread to be the best in the world, and in all our thanksgiving we do not forget the Health Food Company and its valuable products.

JOEL McCOMBER,
56 East Tenth Street, New York."

